# Ketide Synthase (KS) Domain Prediction and Analysis of Iterative Type II PKS Gene in Marine Sponge-Associated Actinobacteria Producing Biosurfactants and Antimicrobial Agents

**DOI:** 10.3389/fmicb.2016.00063

**Published:** 2016-02-12

**Authors:** Joseph Selvin, Ganesan Sathiyanarayanan, Anuj N. Lipton, Naif Abdullah Al-Dhabi, Mariadhas Valan Arasu, George S. Kiran

**Affiliations:** ^1^Department of Microbiology, Pondicherry UniversityKalapet, India; ^2^Department of Botany and Microbiology, Addiriyah Chair for Environmental Studies, College of Sciences, King Saud UniversityRiyadh, Saudi Arabia; ^3^School of Life Sciences, Bharathidasan UniversityTiruchirappalli, India; ^4^Department of Food Science and Technology, Pondicherry UniversityKalapet, India

**Keywords:** glycolipid, lipopeptide, biosurfactant, polyketide synthases, actinobacteria, three-dimensional structure

## Abstract

The important biological macromolecules, such as lipopeptide and glycolipid biosurfactant producing marine actinobacteria were analyzed and their potential linkage between type II polyketide synthase (PKS) genes was explored. A unique feature of type II PKS genes is their high amino acid (AA) sequence homology and conserved gene organization. These enzymes mediate the biosynthesis of polyketide natural products with enormous structural complexity and chemical nature by combinatorial use of various domains. Therefore, deciphering the order of AA sequence encoded by PKS domains tailored the chemical structure of polyketide analogs still remains a great challenge. The present work deals with an *in vitro* and *in silico* analysis of PKS type II genes from five actinobacterial species to correlate KS domain architecture and structural features. Our present analysis reveals the unique protein domain organization of iterative type II PKS and KS domain of marine actinobacteria. The findings of this study would have implications in metabolic pathway reconstruction and design of semi-synthetic genomes to achieve rational design of novel natural products.

## Introduction

Natural products of microorganisms are potential source of bioactives that have been extensively exploited to develop next generation anti-infective drugs proposed by pharmaceutical companies (De Carvalho and Fernandes, 2010). But in recent years, the exploration of marine microorganisms received greater attention due to their complex biosynthetic pathways and potential implications on the development of anti-cancer agents and anti-infectives to combat multi-resistant strains (De Carvalho and Fernandes, 2010). Past few decades the bioprospecting of natural resources and microbial isolates were tremendously increased, however, the leads transformed to drugs are very few (Watve et al., 2001). Perhaps this trend might have led to the exploration of pristine and unexplored bioresources including hydrothermal vents and extreme niches. Marine sponges are sedentary animals harboring more than 40% of microorganisms by volume. Among the marine fauna and flora, marine sponges are potential source of bioactive natural products (Faulkner and Ghiselin, 1983, 1994; Matsunaga and Fusetani, 2003). However, recent deliberations envisage that the sponge derived secondary metabolites are biosynthesized by the associated microorganisms. However, this hypothesis is being remained unproven as sponge-specific bacteria are uncultivable with conventional approaches. Exploration of sponge associated microbial diversity and symbiont-assisted complex biosynthetic pathway of bioactive leads have increased the scope of natural product discovery from marine sponges (Faulkner and Ghiselin, 1983, 1994; Hentschel et al., 2002). Recent developments in genome mining and metagenomics have widely used in the exploitation of such complex biosynthetic pathways of marine natural products. By and large the biosynthetic pathways of polyketides, non-ribosomal peptides, and their derivatives are useful to integrate sponges and their symbiotic biosynthetic machineries. Marine sponges are richest source of polyketide and peptide bioactive molecules. Unlike terrestrial counterparts, sponge-derived bioactive molecules are unique and having specific targeted activities expected for drug leads (Li et al., 2002; Matsunaga and Fusetani, 2003; Montalvo et al., 2005; Montalvo and Hill, 2011). The sponge-derived bioactive peptides are non-ribosomal origin and are modified with unusual amino acids (AAs; Matsunaga et al., 1985).

Polyketide synthases (PKSs) are modular proteins involved in the biosynthesis of complex bioactive molecules through sequential catalytic activities. These enzymes mediate biosynthesis of bioactive molecules with diverse structural complexities by combinatorial use of a specific sequential order of catalytic domains. The tailoring of catalytic domains and AA sequence of these domains are drastically changes with natural bioresources and therefore, the nature and chemical structure of end product is varied between/within the species (Yadav et al., 2009). The mechanism of sequential order and/or selection of catalytic domains remains a major challenge in chemical ecology of secondary metabolite synthesis. The fully dissociable complex of small, discrete mono-functional proteins that catalyze combinatorial synthesis of aromatic polyketides, which is in general termed as type II PKS. In the iterative PKSs, the active site of each catalytic module for tailoring of type II PKS is encoded by a single gene. There is only one set of a hetero-dimeric ketosynthase (KSα–KSβ) and an acyl carrier protein (ACP) that tailored the synthesis of polyketide molecule in a specific order and defined number of cycles to build a polyketide chain (He and Hertweck, 2003). The chain length is maintained through sequential iterative process including cyclisation, reduction, and aromatization steps which are catalyzed by cyclase (CYC), KR, and aromatase (ARO), respectively. In certain group of type II PKSs, the malonyl-CoA ACP acyl transferase (MAT), which catalyzes condensation of acyl transfer between malonyl-CoA and the ACP (Revill et al., 1995). The type II PKSs in general catalyze the biosynthesis of diverse range of multi-functional aromatic polyketides and are mostly restricted among bacteria (Shen et al., 2000). The type II PKSs, such as those responsible for the biosynthesis of the aromatic polyketides actinorhodin (ACT; Fernández-Moreno et al., 1992) and tetracenomycin (TCM), (Bibb et al., 1989; Summers et al., 1992) are composed of three to seven separate mono- or bi-functional proteins, the active sites of which are used iteratively for the assembly and early modification of the polyketide chain.

The KS domain of PKS gene was retrieved from marine actinobacteria producing biosurfactants and antimicrobial compounds. Therefore, this study was aimed to integrate PKS gene in biosurfactant production. Based on the literature, PKS gene can be expected from actinobacteria producing antimicrobial compounds, but PKS gene was not linked with biosurfactant production. A PKS gene possibly encodes biosynthesis of some biosurfactants, being considered as smart biomolecules having the ability to reduce surface and interfacial tension, wider bioactivities and possibly involved in bacterial quorum sensing (Kiran et al., 2015). Biosurfactant production has been reported by our research group in several actinobacteria (Selvin et al., 2009b; Kiran et al., 2010) and they were linked with non-ribosomal peptide synthases (NRPS), PKS (Kiran et al., 2010), and large multifunctional proteins with a modular organization. Biosynthetic pathway of biosurfactants in *Bacillus* and *Pseudomonas* was well-established. However, biosynthetic pathway of biosurfactants produced by marine actinobacteria, in general remains undisclosed. The biosurfactants invariably showed antibiofilm activity without inhibiting the biomass of pathogens tested. Based on *in vitro* experiments, it was found that the biosurfactants produced by marine actinobacteria is having antimicrobial and antibiofilm activity. The PCR amplified KS domain from these actinobacteria envisages the biosynthetic pathway of biosurfactants might have mediated through PKS biosynthetic gene clusters. Therefore, in this study, the *in vitr*o findings are integrated with *in silico* analysis to substantiate the hypothesis that the biosynthesis of biosurfactants produced by marine actinobacteria might have mediated by PKS gene. To date, there are few reports about the interaction between PKS type II gene clusters and biosurfactant production (Kiran et al., 2010). There is no report on marine actinobacteria and their PKS structural diversity related with biosurfactant production. Hence we decided to focus on this aspect with three biosurfactants (MSA10, MSA13, and MSA21; Gandhimathi et al., 2009; Kiran et al., 2010, 2014) and two antagonistic compounds producing (MAD01 and MSI051; Selvin et al., 2009a,b) actinobacterial strains and they were isolated from marine sponges, *Fasciospongia cavernosa* and *Dendrilla nigra*, respectively. *In silico* analysis of PKS gene clusters and modular structure of iterative type II PKS are important tool for designing various experimental approaches toward the combinatorial synthesis of diverse aromatic polyketides. Therefore, present study was aimed to analyze and evaluate the KS domains of iterative PKS gene type II and ketosynthase genes retrieved from marine sponge-associated actinobacteria and their biosurfactant producing ability related to iterative type II PKS gene.

## Materials and Methods

### Microorganisms and PKS Type II Gene Amplification

The actinobacterial strains used in this study were already been isolated from marine sponges, such as *F. cavernosa* (MSA10) and *D. nigra* (MSA13, MSA21, MAD01, and MSI051) collected from southwest cost of India. The 16S rRNA GenBank accession numbers as follows *Nocardiopsis alba* MSA10: EU563352 (Gandhimathi et al., 2009), *Brevibacterium aureum* MSA13: GQ153943 (Kiran et al., 2010), *Brachybacterium paraconglomeratum* MSA21: GQ153945 (Kiran et al., 2014), *Streptomyces* sp. MAD01: GQ246755 (Selvin et al., 2009b), and *Streptomyces dendra* MS1051: EF417875 (Selvin, 2009), respectively. The PKS type II gene was amplified from five actinobacterial strains (MSA10, MSA13, MSA21, MAD01, and MSI051) according to Selvin (2009). The genes encoding PKS were amplified using degenerate primers (**Table 1**). The PCR temperature profile used was 95°C for 3 min, and then followed by 30 cycles at 95°C for 30 s, 56°C for 30 s, and 72°C for 60 s and finally an extension step at 72°C for 10 min. The resultant amplified PCR products were purified and cloned using the TOPO TA cloning kit (Invitrogen) for sequencing.

**Table 1:** PKS type II gene retrieved from marine sponge-associated actinobacteria.

Protein GenBank accession number	Primers	PKS-II amplicon size	Source organism
ACS45380	GCIATGGAYCCICARCARMGIVTGTICCIGTICCRTGISCYTCIAC	579bp	*Nocardiopsis alba* MSA10
ACS45381	GCIATGGAYCCICARCARMGIVTGTICCIGTICCRTGISCYTCIAC	639bp	*Brevibacterium aureum* MSA13
ACS45382	GCIATGGAYCCICARCARMGIVTGTICCIGTICCRTGISCYTCIAC	662bp	*Brachybacterium paraconglomeratum* MSA21
ACV31767	GGIAAYGGITAYGCIMGIGGGTICCIGTICCRTAIGCYTC	519bp	*Streptomyces* sp. MAD01
ABP57802	GGIAAYGGITAYGCIMGIGGGTICCIGTICCRTAIGCYTC	504bp	*Streptomyces dendra* MSI051

### Evaluation of Antibiofilm Effect

The culture supernatant obtained from actinobacterial strains were evaluated for biofilm inhibitory effect against *Vibrio harveyi*. The biofilm was allowed to develop on cover slips and treated with the actinobacterial extracts and incubated for 48 h at 37°C. After incubation the planktonic and spent media were discarded. The cells were washed twice with deionized water air dried and stained with 0.1% acridine orange and examined under confocal laser scanning microscopy (CLSM).

### Determination of Bacterial Cell Viability in Biofilm

Cell viability of the bacteria in the biofilm was assessed using MTT assay as described by Traba and Liang (2011) with necessary modifications. Biofilm of *V. harveyi* was allowed to develop on 96-well plate and treated with 50 μl culture filtrates of the five actinobacterial strains and incubated for 24 h at 37°C. Untreated wells were set as control. After 24 h the bacterial suspension was collected and then treated with 100 μl of phosphate buffered saline and 50 μl of MTT at concentration of 0.3% were added and then incubated for 2 h at 37°C. The MTT solutions were removed and formazan crystals formed were dissolved in 150 μl of DMSO and 25 μl of 0.1 M glycine buffer of pH 10.2. The absorbance was recorded in a microplate reader at 550 nm.

### KS Domain Protein Data Set, Phylogeny Construction, and Domain Structural Analysis

Type II KS domain sequences and ketosynthase gene sequences were translated using sequence manipulation suite^1^ and these deduced AA sequences of type PKS II and ketosynthase were deposited to NCBI-GenBank with the accession numbers of ACS45380–ACS45382 (type II PKS), and ketosynthase bearing following accession numbers ACV31767 and ABP57802. KS domain of type II PKS gene sequences and ketosynthase (Cds) sequences of sponge-associated actinobacteria were retrieved from National center for Biotechnology Information^2^. GenBank accession numbers of these KS domains and ketosynthase sequences were given as GQ153947 (*N. alba* MSA10), GQ153948 (*B. aureum* MSA13), GQ153949 (*Brachybacterium* sp. MSA21), and GQ246762 (*Streptomyces* sp. MAD01), EF520724 (*Streptomyces dendra* MS1051), respectively. The predicted KS domains of all retrieved actinobacterial gene sequences and the PKS type II protein sequences from reference actinobacteria were aligned by CLUSTAL W2^3^ and translated deduced AA sequences were verified using the NCBI-BLAST^4^ search with expected value set to the default value of 10 was performed using the protein sequences of *N. alba, B. aureum, Brachybacterium* sp. MSA21*, Streptomyces* MAD01 and *S. dendra*, respectively, and the various sequences against 138 complete eubacterial and 20 complete archaebacterial genomes. Phylogenetic tree of the deduced AA sequences of PKS II segments and ketosynthase genes were generated using neighbor-joining method through MEGA programs (Kumar et al., 2004). KS domain phylogeny was based in the prediction of putative enzymes of identical or nearly identical biochemical function. The type of KS was identified based on the top BLAST match in the reference data set. NCBI CDD search, SEARCPKS and Motif scan were performed to derive the existence of significant domains and their organization. Comparative analyses of KS domains of five subject organisms were performed with known polyketide producers and with the structure of polyketides using NCBI CDD and SEARCHPKS, respectively. The AA composition was also predicted to substantiate the function of type II PKS and ketosynthase of our interest.

Profile Hidden Morkov Model (HMM) analysis was carried out by HMMER package. The available three (*Nocardiopsis*, *Brevibacterium, Brachybacterium*) actinobacterial KS dataset was analyzed, whether these domains are modular or iterative KS domains. All these three iterative KS domains of PKS type II gene clusters of actinomycetes were modeled using comparative modeling approach. Threading analysis was carried out using a local version of threader package^5^ to identify the structural templates for modeling of actinobacterial KS domains. The remaining two KS domains (from *Streptomyces* MAD01 and *S. dendra* MSI051) have been modeled using fatty acid KAS structure as template (*Escherichia coli* KAS I), which show only about 40% sequence identity with polyketide KS domains. Even the sequence identity was lesser between the target and template, the two KS proteins structures can be reliable and they adopt similar structure. The secondary structures of type II PKS and ketosynthase domains of 3D models were created using a (PS)^2^ is an automated homology modeling server (Chen et al., 2006). The (PS)^2^ combines PSI-BLAST, IMPALA, and T-Coffee in both template selection and target-template alignment. The final three dimensional structures were built using the modeling package MODELLER.

## Results And Discussion

### The Nature of KS Domains of Type II PKS and Ketosynthase

**FIGURE 1:**
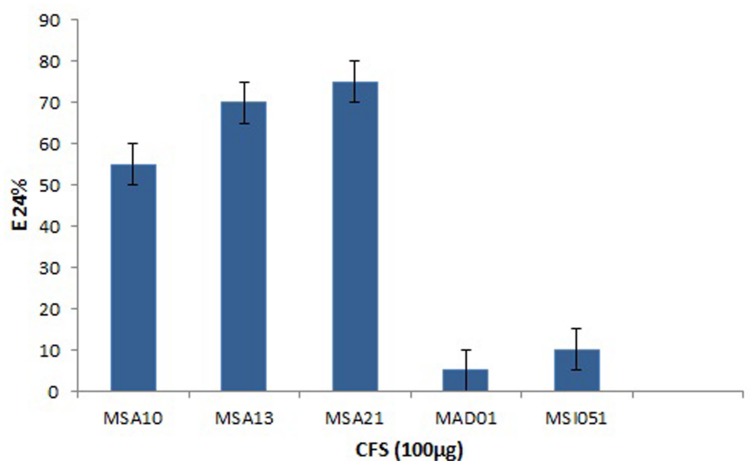
**Emulsification index of biosurfactants produced by marine actinobacteria**.

The actinobacterial isolates from marine sponges were screened for biosurfactant activity using emulsification index (E_24_) as per Kiran et al. (2010). Among the five actinobacteria MSA10, MSA13, and MSA21 were potential producer of biosurfactants (**Figure 1**). The active moieties were identified from GCMS data. The active moieties of MSA10, MSA13, and MSA21 were evidenced as biosurfactant molecules, but the moieties of MAD01 and MSI051 were not related with biosurfactants (**Table 2**). The antimicrobial moiety of MAD801 was identified as cyclohexane carboxylic acid hexyl ester. It was reported that cyclohexane carboxylic acid is a moiety of the antifungal polyketide ansatrienin A (Patton et al., 2000).

**Table 2:** 3D structures of active moieties identified from GC-MS data.

Strain name	Compound	3D structures of active moieties detected from GS-MS data	
MSA10	Lipopeptide	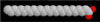	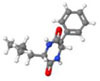

MSA13	Lipopeptide	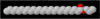	Gly–Gly–Leu–Pro–

MSA21	Glycolipid	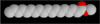	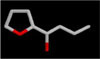

MAD01	Cyclohexanecarboxylic acid	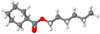	

MSI051	2,5-Piperazinedione, 3,6-Bis Phenylmethyl	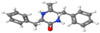	

Sponge-associated actinobacteria: i.e., *N. alba, B. aureum*, and *Brachybacterium paraconglomeratum* beared the type II PKS (GQ153947, GQ153948, and GQ153949, **Table 1**). The KS domain of these gene segments were translated into AAs counts, viz; 191, 212 and 220, respectively. All these KS domains encodes the condensation enzymes (cds), which catalyzes (decarboxylation or non-decarboxylation) Claisen-like condensation reaction, and the KS domains sharing the strong structural similarities are involved in the synthesis and degradation of fatty acids.

**FIGURE 2:**
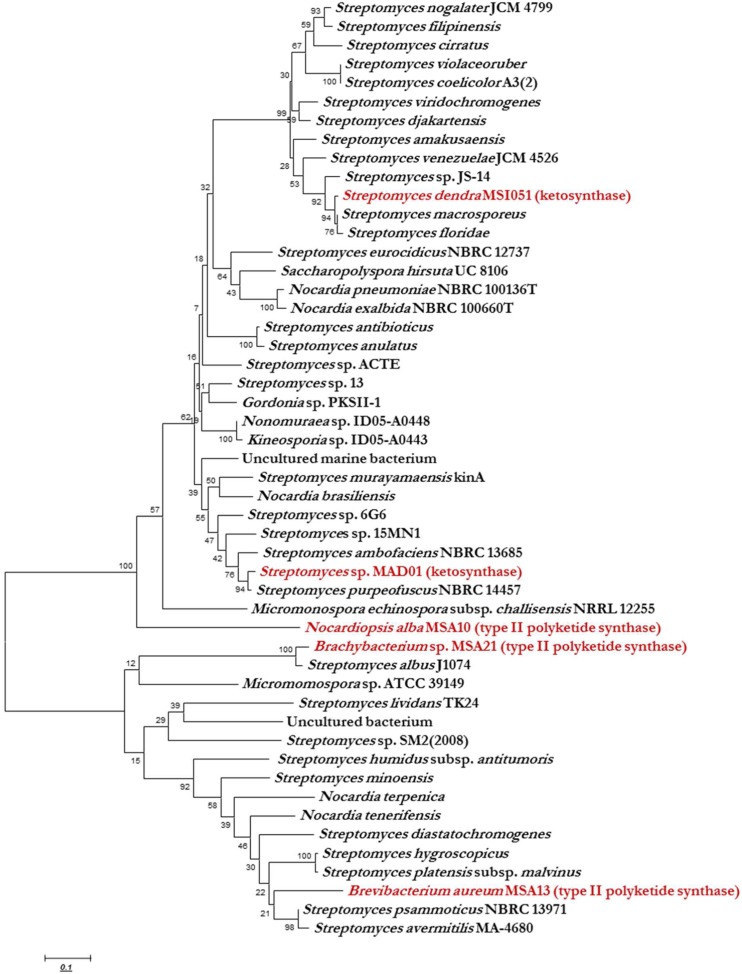
**Phylogenetic analysis (MEGA 5.0) of ketosynthase regions with respect to the diverse range of ketosynthase domains, including iterative types II, modular PKS, and KS domain.** The phylogenetic trees were constructed using bootstrapping and the neighbor-joining rules.

KS domain of PKS gene is the most conserved catalytic domain and is involved in the tailoring PKS molecule by catalyzing the chain condensation step. We have performed *in silico* analysis to identify KS domain counterparts from modular and iterative PKSs and other PKS families. The analyzed domains are separated into distinct clusters in a phylogenetic tree (**Figure 2**). Based on HMM by the HMMEP package, three actinobacterial KS domain of type II PKS genes were analyzed and the results show that these three isolates contains iterative PKS gene and this outcome provides potential in genome sequencing efforts for the identification of novel PKS genes. Iterative condensation steps play a vital role in biosynthesis by PKS proteins and phylogenetic analysis of iterative KS domains inferred that the clustering of iterative PKS gene sequence is highly correlated with the number of iterations they perform. From this study, we suggest that marine sponge associated actinobacterial community predominantly possesses the iterative KS domain of type II PKS rather than modular type I PKS or NRPS-PKS hybrids. The type II PKS from three different genera is characterized to study and understand their function and diversity. The isolation and identification of PKS with different enzymatic activity in marine actinobacteria has been reported, as well as the occurrence of PKS gene families in a community (Kim and Fuerst, 2006). This is the first report on the *in silico* analysis of iterative type II PKS of sponge-associated actinobacteria. Recent literature (Kiran et al., 2010, 2015) evidenced that these actinobacteria are potent biosurfactant producers with antimicrobial activity (lipopeptide and glycolipid derivatives). The present *in silico* analysis revealed that these isolates possessing iterative domains (**Figure 2**) type II PKS genes and it can be hypothesized that the antimicrobial biosurfactants synthesis might be mediated by iterative type II PKS genes. Another group of actinobacterial antibiotics producers from the marine sponge *D. nigra* such as *S. dendra* MSI051 (Selvin, 2009) and *Streptomyces* sp. MAD01 (Selvin et al., 2009b) were included in the analysis. Their partial ketosynthase genes were retrieved from GenBank (GQ246762 and EF520724) with 519 and 504 bp encoding 173 and 168 aa, respectively (**Table 1**).

### Antibiofilm Effect Against *Vibrio*

**FIGURE 3:**
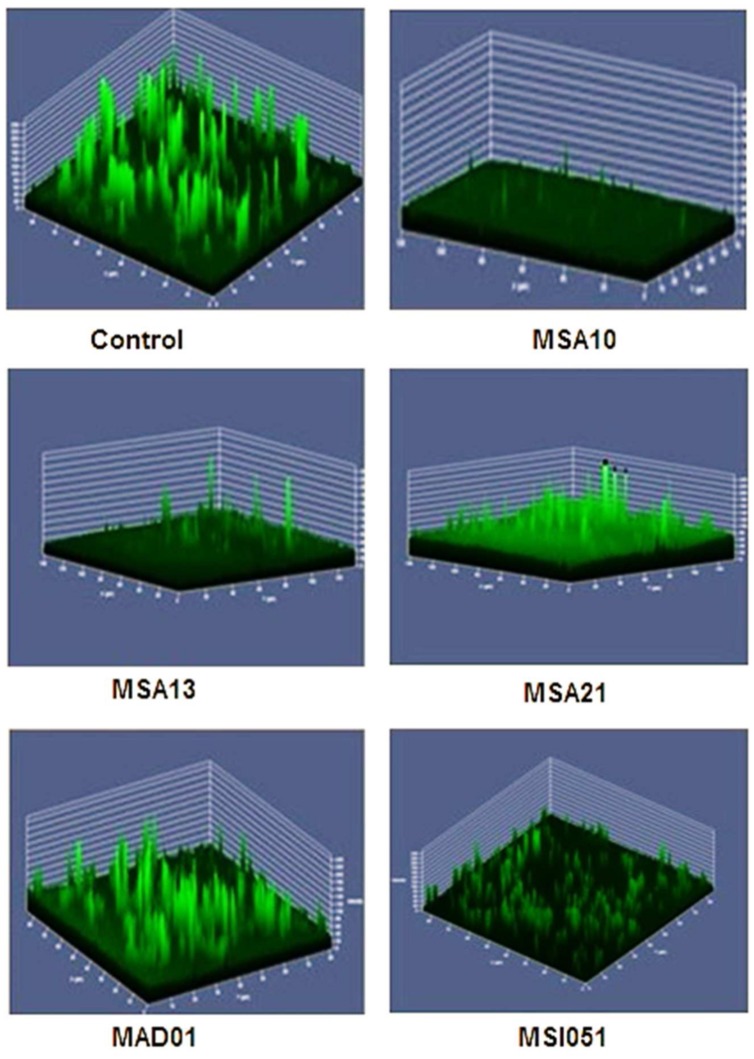
**Antibiofilm activity of cell free supernatants from marine actinobacteria.** The pre-formed biofilm on glass slides was treated for 24 h with 150 μg each of freeze-dried cell free supernatant (CFS). Untreated biofilm (control) and remaining biofilm of *V. harveyi* treated after CFS on glass slides were stained with 0.1% acridine orange and observed in CLSM (LSM 710, Carl Zeiss).

Antibiofilm effect of the culture supernatant was well noticed by CLSM. The culture supernatant inhibits the biofilm formation *of V. harveyi*. Among the extract used the lipopeptide producer MSA10 and MSA 13 inhibit the biofilm formation by 80% compared to the other actinobacterial extracts (**Figure 3**). The antibiofilm effect may be due to the biosurfactant production mediated by PKS gene.

### Cell Viability in Biofilm

**FIGURE 4:**
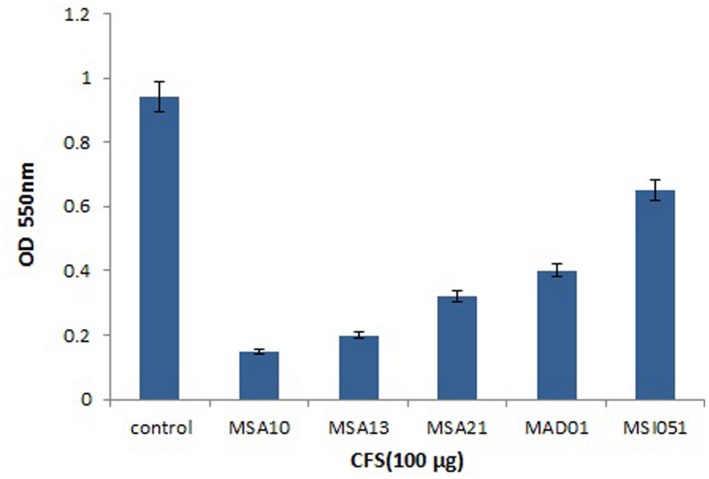
**Cell viability of *V. harveyi* treated with CFS of marine actinobacterial cultures**.

The viability of the cells were reduced by adding the biosurfactants as shown in **Figure 4**. When compared to the control the extracts from MSA 10 and MSA 13 inhibits the viability of *Vibrio* cells by more than 80%, followed by MSA 21 by 70%.

### Domain Architecture and Homology Modeling of Iterative Type II PKS

*In silico* analysis of Type II PKS and ketosynthase unveiled an unprecedented organization of various domains encoding discrete ketoacyl synthase (KAS) and thiolase, PKC, CK2, and ACP some are lacking an ACP. Certain polyketides undergoes non-iterative biosynthesis which involves a novel type II PKS that acts directly on acyl CoA substrates. These results demonstrate the capability of nature’s in designing complex bioactive compounds and suggest new methods for PKS design and engineering through synthetic biology approaches to expand the scope and diversity of polyketide library. The structural diversity of PKS would ultimately help in searching for PKS with novel chemistry for combinatorial biosynthesis (Shen and Kwon, 2002). All the proteins studied here are found to have potential KS domains which catalyze the polyketide chain elongation step. In the beginning of chain elongation, an enzyme intermediate is formed between the growing polyketide chain and the thiol of its active site Cys. Then condensation reaction occurs with the methylmalonyl-ACP or malonyl-ACP co-substrate (Shen, 2003).

The phylogenetic trees were constructed using bootstrapping and the neighbor-joining rules.

**FIGURE 5:**
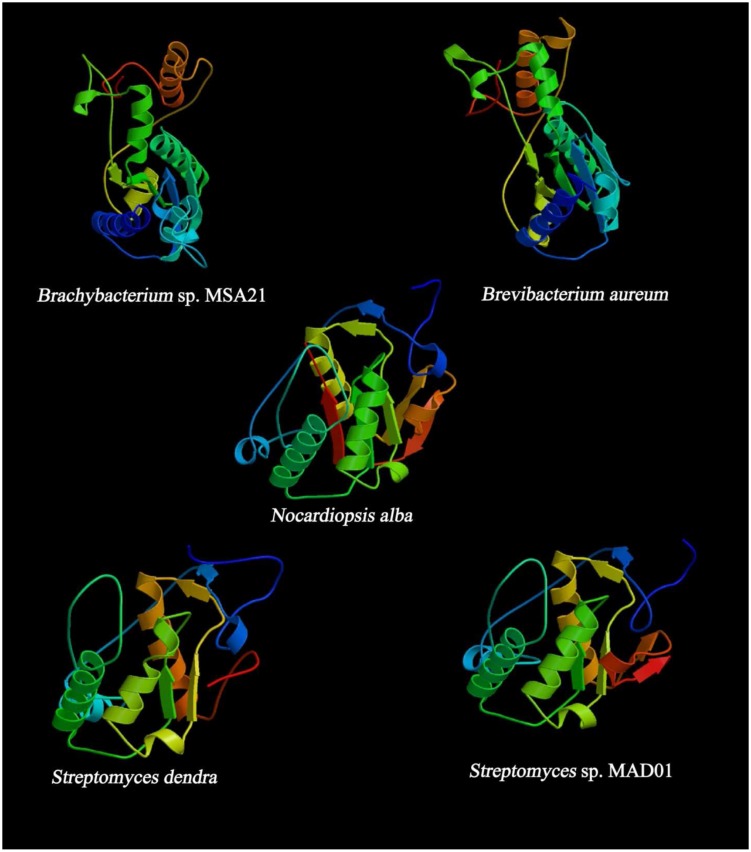
**Predicted secondary structure of KS domain using (PS)^2^: Protein Structure Prediction Server**.

Analysis evidenced that the PKS sequence retrieved from *N. alba* and *B. aureum* are having ACP domain, i.e., beta-ketoacyl-ACP synthase and beta-KAS (**Figure 5**). KASs are involved in the elongation steps in the pathway of fatty acid biosynthesis. KAS III is involved in the catalysis of the initial condensation and KAS I and II are responsible for elongation steps by Claisen condensation of malonyl-ACP with acyl-ACP. Remaining three protein sequences lack ACP, some non iterative type II PKSs lack ACP, utilize acyl CoAs as substrates for macrotetrolide biosynthesis. It was reported that the PKSs are using ACP to activate the acyl CoA substrate and channel the polyketide intermediates (Shen, 2003).

Outside of the module, the beta-KAS domains are dimeric. However, the number of domains within the module is dimeric still remains to be established (Tsai et al., 2001, 2002; Broadhurst et al., 2003). Perhaps every enzyme within the module made contacts across the *ser* and *cys*, ACP suppose to diffuse farther than the peptide linkers on each side would permit (Keatinge-Clay and Stroud, 2006). The deduced quaternary structure of the proteins indicates a surprising configuration which is homologous to many PKS genes that are capable of synthesizing active polyketides. Even alignment of KS domains of our sequence of interest shows 53–62% of similarity with structures like amphotericin, ACT, epothilone, meagalomycin, myxalomycin, and rifamycin.

Most of the KS are dimeric with active site at the interface of dimer and type II PKS probably functions by making contact across the twofold axis and the active sites of KS are accessible to ACP (Keatinge-Clay and Stroud, 2006). In the present findings, we observed the PKC domain is common in all the protein sequences and lack AT domain. The analysis showed the chances of inactive enzymes within the modules may perform some important functions. The ACP module is bound by peptide linkers on both ends, and this module can pass between each enzyme in the module as well as the next KS or thiolase C and N terminal (Perham, 2000). The linkers helps to prevent a polyketide from interacting with enzymes and contribute little translational freedom to the polyketide compared to the peptide linkers on both ends of ACP. Thus helps in the biosynthesis of polyketides (Keatinge-Clay and Stroud, 2006). The interaction of ACP with the KS domain facilitate to docks in a deep groove which is formed by the interaction of the KS, PKC, and the other linker, thereby implicating both the PKC and the thioesterase linker in functional KS-ACP recognition (Liu et al., 2002). The KS domain of type II PKS (*N. alba*) ACS45380.1 was closely related to those of ACT PKS. Type II PKS (*N. alba*) consists of seven structural domains includes Asn Glycosylation, CK2 Phospho site, PKC, Tyr Phosphosite, ACP and KAS (49–161 AA residues) which shares 59% similarity with ACT polyketide putative beta-KAS 2 which contains eight chains, out of which two chains are homologous to our PKS protein which are chain A: beta-KAS/acyl transferase and chain B: ACT polyketide putative beta-KAS 2. The synthesis of aromatic polyketides are mostly begins with the formation of a polyketide chain (Keatinge-Clay and Stroud, 2006). The polymeric chains of type II PKS are tailored by the heterodimeric ketosynthase-chain length factor (KS-CLF). KS-CLF is the homolog of KS domain of type II PKS of *N. alba* which regulates chain length by catalyzing both chain initiation and elongation. Exploration of the mechanistic details of this central PKS polymerase may support designing and reconstruction of pathways being invented on synthetic biology platforms. This protein was structurally elucidated with four alpha helix and seven beta sheets. And it is slightly acidic composed of 39.79% of aliphatic (G,A,V,L,I), 18.32% of Acidic (B,D,E,N,Q,Z), 15.18% of basic (K,R,H), 3.66% of sulfur (C,M), 3.66% of aromatic (F,W,Y), and 12.04% of aliphatic hydroxyl (S,T).

Type II PKS of (*B. aureum)* ACS45381.1 is sequentially identical to type I ketosynthase (*Streptomyces* sp. T12-208) ACR61389.1. Structurally it is similar to the human fatty acid synthase (FAS), a modular enzyme involved in the metabolism of fatty acids and a drug target of antineoplastic and anti-obesity agents. Detailed structural study on human FAS has been limited due to its size and flexibility. Large part of human FAS that encompasses the tandem domain of beta-KAS is closely related to the KS domain of *B. aureum.* The KS domains are appear as the canonical dimer, and its substrate-binding site differs from that of bacterial homologs but is similar to type II PKS of *B. aureum*.

According to domain analysis, the PKS is a multi-domain protein consists of 14 domains includes ASN glycosylation, PKC, CK2, beta KAS, ACP synthase III, thiolase C and N terminal, and KAS C terminal domains. The position of KAS domain is 1–151 and 159–212 AA residues. The AA composition of the protein is predicted with 48.58% of aliphatic (G,A,V,L,I), 5.19% of aromatic (F,W,Y), 2.83% of sulfur (C,M), 9.43% of basic (K,R,H), 16.51% of acidic (B,D,E,N,Q,Z), and 14.15% of aliphatic hydroxyl (S,T). Type II PKS (*Brachybacterium* sp. MSA21) ACS45382.1 is identical to type I PKS of *Streptomyces* sp. and structurally proposed to contain five domains as follows KAS C and KAS N, myristyl site, PKC, and thiolase. The tertiary structure of the protein depicts six alpha helix and six beta sheets and composed of 50.45% of aliphatic (G,A,V,L,I), 5% of aromatic (F,W,Y), 2.73% of sulfur (C,M), 11.36% of basic (K,R,H), 13.18% of acidic (B,D,E,N,Q,Z), and 13.18% of aliphatic hydroxyl (S,T) AAs.

The position of the KS domain is 1–159 and 167–220 AA residues. In PSI- BLAST, PKS is predicted to have a structure similar to chain A, the ACT ketosynthase chain length factor since 73% identity, the *E*-value: 6.61*e* – 67, bit-score: 256, aligned-length: 173, this protein is structurally related to ACT ketosynthase and proposed to be rich in acidic and aliphatic AA residues, since the ligands may be acetyl group/magnesium ion/sodium ion. Six alpha helix and five beta sheets., and the composition is 41.04% of aliphatic (G,A,V,L,I), 6.94% of aromatic (F,W,Y), 3.47% of sulfur (C,M), 13.87% of basic (K,R,H), 19.08% of acidic (B,D,E,N,Q,Z), and 10.40% of aliphatic hydroxyl (S,T). The ketosynthase (*Streptomyces dendra*) ABP57802.1 found to have seven domains includes CKII phosphorylation site, PKC, KS C terminal and N terminal, phage tail fiber repeat and the AA composition is 42.26% of aliphatic (G,A,V,L,I), 4.76% of aromatic (F,W,Y), 4.17% of (sulfur C,M), 13.69% of basic (K,R,H), 18.45% of acidic (B,D,E,N,Q,Z) and 11.90% of aliphatic hydroxyl (S,T). The structural configuration presents six alpha helix and four beta sheets and mimics the structure of chain A. The ACT ketosynthase CLF with the values as follows, *E*-value: 1.00*e* – 72, bit-score: 258, aligned-length: 191, and identity to query: 67%. The PSI-BLAST shares 99% of similarity with doxorubicin PKS (*E*-value 4*e* – 75), 3-oxoacyl-ACP synthase I (*Streptomyces avermitilis* MA-4680), putative ketosynthase of *Streptomyces antibioticus* with (*E*-value 4*e* – 60), and granaticin polyketide putative beta-KAS 1 of *Streptomyces hygroscopicus* ATCC 53653 (*E*-value 3*e* – 60). KS domain analysis of type II PKS and ketosynthase was performed using PSI BLAST and MEGA (CLUSTAL W2) to highlight the unique conservative motif of each protein. The strain *B. aureum* shares specific motif with BAH67362.1 (PKS *Streptomyces minoensis*) denoted as “VDTACSSSLVALHLAAQALRSG.” Comparative analysis of KS domain of *Brachybacterium* sp. MSA21 exhibit the presence of unique motif PQQR(H)L in all the reference sequences which are capable of synthesizing cirramycin (BAH67190), minomycin (BAH67362), maridomycin (BAH67036), an anticancer compound (BAH67464), and platiomycin (BAH67144). This is the first report on the possible structural diversity mediated by type II PKS in *Brachybacterium* sp. MSA21.

### KS Domain Phylogeny

Ketosynthase domain phylogeny was used to infer the phylogeny of type II PKS and ketosynthase. Phylogenetic analysis showed that the sponge associated actinobacterial sequences of PKS II genes and KS fragments were matched to conserved regions of previously characterized functional domains of other PKS I, II, and ketosynthase proteins. The KS domain of *Brachybacterium* and *Brevibacterium* showed a unique clustering, found KS domain of *Brevibacterium* clustering between two *Streptomyces* group and each group having two isolates and they possess high similarities among them like 100 and 98, respectively, but having less homology with *B. aureum* (**Figure 2**). The *Brachybacterium* was closely clustering with *S. albus* J1074 with 100% similarity. *N. alba* was not clustered with any actinobacterial KS domain since it was having the unique identity with KS domain of *Streptomyces* MAD01 and *S. dendra*. KS domain of *Streptomyces* MAD01 showing 97% of similarities with *S. purpeofuces* NBRC 14457 and *S. dendra* was clustering between *Streptomyces* sp. JS-14 and *S. macrosporeus* with 98 and 97% similarity, respectively. From the cluster analysis, we observed that two different marine sponge-associated actinobacteria possessing the identical KS domains of iterative PKS.

The major aim of this study was to find out the gene diversity of the PKS II and ketosynthase in two marine sponge associated actinobacterial polulation. The KS gene diversity could be useful to understand the evolution pattern of actinobacteria in the marine sponges, mode of interaction between sponge and associated microbes (Selvin, 2009) and chemical diversity of PKS II in marine sponge. Phylogenetic analysis of iterative PKS sequences is highly correlated with the number of iterations they performs. The PKS gene analysis provide a new insights that the poorly studied genera, such as *Brevibacterium* and *Brachybacterium* represent the KS genes which proves the unexplored resource for natural-product discovery. Conversely, the nearly ubiquitous detection of PKS genes in *Streptomyces* and *Nocardiopsis* envisages the possibility similar kind of natural products, but in reality the compounds are expected to be highly complex with diverse bioactivities (Selvin, 2009; Selvin et al., 2009b). To overcome these challenges, KS domain of PKS genes retrieved and analyzed in this study. KS domains tend to cluster phylogenetically based on the secondary metabolites of the actinobacterium from which the gene was retrieved. The active KS domains predictions could be based simply on the analysis of around 500 bp regions of KS domain from single PKS gene. The level of KS sequence domain in the iterative biosynthesis of natural products needs to be determined. The level of KS domain in strains may differ as it depends on the rate of sequence evolution, niche selectivity, host evolution pattern, and the time of pathways have been isolated in the respective genomes. It is also of interest that the three KS sequences associated with iterative type II PKS pathways were widely distributed among diverse taxonomic groups. The fact that all these KS domain sequences display relatively low levels of identity to the ketosynthase domain of *Streptomyces* MAD01 and *S. dendra* suggests that they are not associated with the production of iterative type of PKS domains. The mixed clustering of different sponge associated KS domains already been documented here for the first time we are reporting the evolutionary relatedness of KS domains of type II PKS and ketosynthase from *D. nigra* and *F. cavernosa* isolates. According to recent literatures, the PKS genes and their products exhibit novel insights in antimicrobial drug discovery (Selvin, 2009; Sasso et al., 2014; Wang et al., 2014). KS domain of type II PKS phylogeny is also highly need to know their relationship and structural diversity.

### Potential Linkage Between Iterative Type II PKS Gene and Lipopeptide and Glycolipid Biosurfactant Production in Marine Sponge-Associated Actinobacteria

The marine sponge-associated bacteria have been recognized as rich source of biological macromolecules that are of potential interest to various industrial sectors (Kiran et al., 2015). Study reports evidenced that marine actinobacteria are unexplored resource for biosurfactant production. In this study, three actinobacterial strains (MSA10, MSA13, and MSA21) isolated from marine sponge were able to produce lipopeptide and glycolipid biosurfactants, respectively and showed positive for type II PKS gene. Two actinobacterial strains (MAD01 and MSI051) from *D. nigra* failed to produce biosurfactants but has the capability to synthesis polyketide based antagonistic compounds. There is an existing evidence for the synthesis of lipopeptide biosurfactant in *Bacillus subtilis* by NRPSs or hybrid PKS/NRPSs (Ongena and Jacques, 2008). These modular proteins in marine sponge associated microbes are responsible for the biosynthesis of several bioactive metabolites. They are mega-enzymes structured by iterative functional units called modules catalyzes various condensation, reduction, transferase reactions leading to polyketide and peptide transformation for the synthesis of biosurfactant. The positive strains display biosurfactant activity and, significantly, iterative type II PKS domain gene fragments, indicating the existence of a PKS gene cluster associated with biosurfactive compound biosynthesis. The present study reveals that the actinobacteria are a rich source of bioactive compounds and biosurfactant, and also represent the unrecognized group of organisms having type II PKS systems for polyketide biosynthesis. In this study, the bacterial motility was also checked for the surfactive compound production (data not shown). Bacterial motility mechanisms, comprising swimming, swarming, and twitching, are known to have significant roles in biofilm formation, colonization, and the subsequent expansion into complete organized surface populations. All the actinobacterial strains showed positive in swimming, swarming, and twitching motility assays which indicate that these strains possess biofilm forming ability. The strains with increased swimming motility also possess good swarming ability. Current research evidenced that the strain *Streptomyces* sp. MAD01 possess good biofilm forming capacity as well as antimicrobial activity against test organisms. It also proves that a ketosynthase type II PKS system is responsible for the biosynthesis of the antagonistic compounds in marine actinobacteria. Based on the present findings, the production of biosurfactants might be linked with type II iterative PKS gene cluster and the synthesis of biosurfactant by the sponge-associated actinobacteria might have significant role in the chemical ecology of host sponge (Kiran et al., 2015). However, the hypothesis has not been tested in controlled *in vitro* and *in vivo* experiments. Based on the functions of biosurfactants including antibacterial/antibiofilm activity, the biosurfactants may play a role in host defense fouling processes (Gandhimathi et al., 2009; Kiran et al., 2014). Therefore, explorations of marine sponge-associated actinobacteria for the lipopeptide and glycolipid biosurfactant production will have wider applications in industrial processes, bioremediation, and enhanced oil recovery.

## Conclusion

In the current study, the iterative nature of actinobacterial type II PKS was proved by HMM profile. The domain architecture of *N. alba* and *B. aureum* have the potential of constructing “minimal PKS” and the later species share the specific motif “VDTACSSS” with *S. minoensis*. Both strains displayed PKS domains structurally similar with encoding ACT. *S. dendra* is found to have a unique repeat called phage tail fiber repeat which is responsible for altering the host specificity of secondary metabolites through protein–protein interaction. The other three actinobacterial strains *Brachybacterium* sp., *Streptomyces* sp., and *S. dendra* lack ACP results in inactive minimal PKS or may act non-iteratively. This study also provides a new insight on the KS genes of *Brevibacterium* and *Brachybacterium* proving that marine resources are still largely unexplored for natural-product discovery. In these regards, *in silico* gene mining is quite useful for prospecting novel metabolites produced by marine sponge endosymbionts. Further *in vitro* studies are needed to design novel natural products using a biosynthetic engineering approach.

## Author Contributions

JS and GSK designed the experiments, GS performed *in silico* analysis, ANL performed *in vitro* assays, NAA and MV performed data analysis and interpretation, GSK, GS, and JS written the manuscript.

## Conflict of Interest Statement

The authors declare that the research was conducted in the absence of any commercial or financial relationships that could be construed as a potential conflict of interest.

## Footnotes

1 http://www.bioinformatics.org/sms2/

2 www.ncbi.nlm.nih.gov/GenBank

3 http://www.ebi.ac.uk/Tools/msa/clustalw2

4 www.ncbi.nlm.nih.gov/BLAST

5 http://bioinf.cs.ucl.ac.uk/psipred/
